# Supplementary material for: Identification of photoperiod-induced specific miRNAs in the adrenal glands of Sunite sheep (Ovis aries)
Source: Front Vet Sci. 2022 Jul 22;9:888207. doi: 10.3389/fvets.2022.888207 (PMC9354845; doi:10.3389/fvets.2022.888207)
Supplement: Supplementary file 1 [file Data_Sheet_1.ZIP › Supplementary file/miRDeep2 file.zip!/miRDeep2 file/Novel_Structure/SPLP42A2/Novel_621.pdf]

Provisional ID	:	NC_019471.2_27998
Score total	:	163.7
Score for star read(s)	:	3.9
Score for read counts	:	155.6
Score for mfe	:	2.6
Score for randfold	:	1.6
Score for cons. seed	:	
Total read count	:	317
Mature read count	:	316
Loop read count	:	0
Star read count	:	1

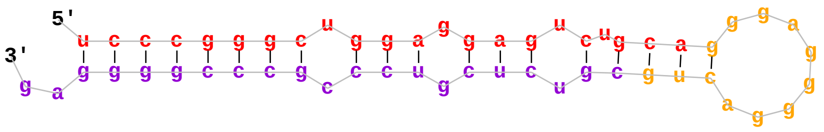

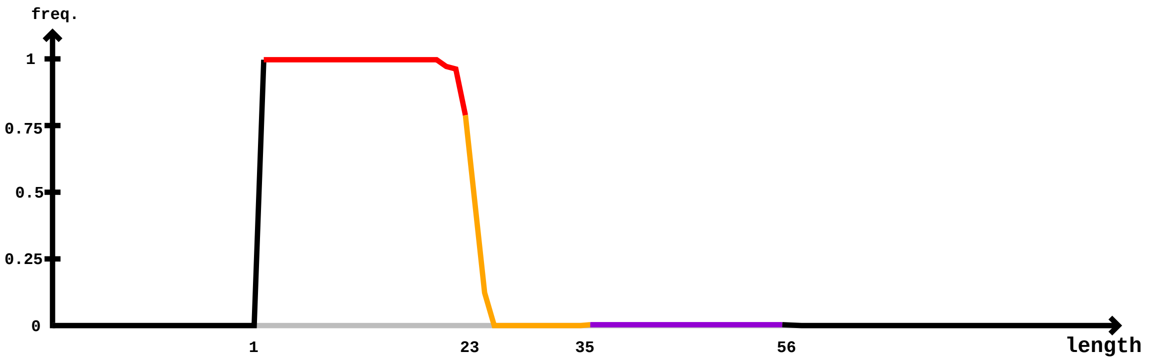

Mature **Star**

```
5'- ggaccugggcaggggcgggguccccgggcuggaggagucugcagggaggggacugcgucucgucccgcccggggagaccgccccuugccgcucccaggacgguggauagc   -3'   obs
    ggaccugggcaggggcgggguccccgggcuggaggagucugcagggaggggacugcgucucgucccgcccggggagaccgccccuugccgcucccaggacgguggauagc         exp
    .......((((((((((..((((((((.(((.(((.(.((((........))))).))).))).))))))))...))))))).))))(((.(((......)))..)))   reads   mm   sample
    ....................ucccgggcuggaggagucu......................................................................   8       0    seq
    ....................ucccgggcuggaggagucug.....................................................................   3       0    seq
    ....................ucccgggcuggaggagucugc....................................................................   55      0    seq
    ....................ucccgggcuggaggagucugca...................................................................   143     0    seq
    ....................ucccgggcuggaggagucugcag..................................................................   68      0    seq
    ....................ucccgggcuggaggagucugcagg.................................................................   39      0    seq
    ......................................................cgucucgucccgcccggggag..................................   1       0    seq
```
